# Novel Insight into the Genetic Context of the *cadAB* Genes from a 4-chloro-2-methylphenoxyacetic Acid-Degrading *Sphingomonas*

**DOI:** 10.1371/journal.pone.0083346

**Published:** 2013-12-31

**Authors:** Tue Kjærgaard Nielsen, Zhuofei Xu, Erkin Gözdereliler, Jens Aamand, Lars Hestbjerg Hansen, Sebastian R. Sørensen

**Affiliations:** 1 Department of Geochemistry, Geological Survey of Denmark and Greenland, Copenhagen, Denmark; 2 Section for Microbiology, University of Copenhagen, Copenhagen, Denmark; University of Kentucky College of Medicine, United States of America

## Abstract

The 2-methyl-4-chlorophenoxyacetic (MCPA) acid-degrader *Sphingomonas* sp. ERG5 has recently been isolated from MCPA-degrading bacterial communities. Using Illumina-sequencing, the 5.7 Mb genome of this isolate was sequenced in this study, revealing the 138 kbp plasmid pCADAB1 harboring the 32.5 kbp composite transposon Tn6228 which contains genes encoding proteins for the removal of 2,4-dichlorophenoxyacetic acid (2,4-D) and MCPA, as well as the regulation of this pathway. Transposon Tn6228 was confirmed by PCR to be situated on the plasmid and also exist in a circular intermediate state - typical of IS3 elements. The canonical *tfdAα*-gene of group III 2,4-D degraders, encoding the first step in degradation of 2,4-D and related compounds, was not present in the chromosomal contigs. However, the alternative *cadAB* genes, also providing the initial degradation step, were found in Tn6228, along with the 2,4-D-degradation-associated genes *tfdBCDEFKR* and *cadR*. Putative reductase and ferredoxin genes *cadCD* of Rieske non-heme iron oxygenases were also present in close proximity to *cadAB*, suggesting that these might have an unknown role in the initial degradation reaction. Parts of the composite transposon contain sequence displaying high similarity to previously analyzed 2,4-D degradation genes, suggesting rapid dissemination and high conservation of the chlorinated-phenoxyacetic acid (PAA)-degradation genotype among the sphingomonads.

## Introduction

2-methyl-4-chlorophenoxyacetic (MCPA) and 2,4-dichlorophenoxyacetic acid (2,4-D) are widely used phenoxyacetic acid herbicides that are occasionally found in the Danish groundwater aquifers [1]. The complete degradation of 2,4-D and MCPA have been described with the first step being performed by the products of the genes *tfdA*, *tfdAα* or *cadAB* [2]–[5]. The product of the first catabolic step is then further degraded by the products of genes *tfdBCDEF* to finally yield β-ketoadipate, which is degraded in the central metabolism [6]. Most attention has been paid to *tfdA* and *tfdAα* [3]–[5], [7], while *cadAB* has been less described [2], [8], [9]. A recent study on MCPA degradation in soil microcosms showed that both the *cadA* and *tfdA* genes are simultaneously expressed during MCPA degradation in soil [10], indicating that additional knowledge should be gathered on the *cadAB* genes if they are to be compared with the well-studied *tfdA* gene.

Phenoxyacetic acid (PAA) herbicide degraders are generally divided into three categories [5], [9], [11]. The first group consists of β- and γ-proteobacteria, which are characterized by the involvement of the *tfdA* gene as the first step in the degradation of phenoxyacetic acid herbicides. Within group I degraders, three further subclasses have been described, based on the similarities of the *tfdA* sequences (class I–III) [12]. Group II consists of α-proteobacteria that are phylogenetically closely related to *Bradyrhizobium* sp. Remarkably, representatives of this group were initially isolated from pristine environments in Canada, Chile and Hawaii [11]. This group was shown to possess a *tfdA*-like gene, termed *tfdAα*, which is 56–60% similar to the representative *tfdA* gene of the canonical group I degrader *Cupriavidus pinatubonensis* JMP134 [5]. Group III degraders are α-proteobacteria closely related to the *Sphingomonas* genus, also harboring the *tfdAα* gene [9].

The CadAB catabolic proteins have been shown to perform the same initial step in 2,4-D degradation as TfdA. However, CadAB are subunits of a Rieske non-heme iron oxygenase, making its mode of action quite different from the α-ketoglutarate-dependent dioxygenase action of TfdA [2], [3]. CadAB of *Bradyrhizobium* sp. HW13 shows homology to the benzoate dioxygenase BenAB and the 2,4,5-T catabolic protein TftAB [2]. The *cadA* gene has been found in members of both group II and III PAA-herbicide degraders [2], [8], [9], [13]. Some of these bacteria also have the *tfdAα* gene, displaying a dual-system of degradative genes [9].

Previously, a gene cluster from *Sphingomonas* sp. 58-1 containing the genes *cadAB*, was cloned into an *E. coli*, resulting in 2,4-D degradation in this host. Mutational analysis showed that both *cadA* and *cadB* were essential for 2,4-D degradation [8]. In this study, the MCPA- and 2,4-D-degrading bacterium *Sphingomonas* sp. ERG5, recently isolated from the MCPA-degrading bacterial communities described in [14], had its genomic content shotgun sequenced using Illumina-sequencing. This bacterium was shown to have a transposon, harboring *cadAB* as well as other PAA-herbicide catabolic and regulatory genes, situated on a plasmid containing genes for conjugative transfer.

## Methods

### DNA extraction and Illumina sequencing

DNA was extracted from strain ERG5 grown on R2A (Difco™) plates at 20°C supplemented with 100 mg/l of MCPA (Fluka Chemie AG), using the PowerLyzer™ UltraClean® Microbial DNA Isolation kit (MOBIO). A swab of bacteria from a single colony was picked for DNA extraction. Cell lysis was performed using the FastPrep-24 instrument (MP Biomedicals) for 3×30 seconds at 4 m/s with samples chilled on ice between runs. The extracted DNA was quantified using a QUBIT® 2.0 fluorometer (Invitrogen) and was measured to be 7.82 ng/µl. The low quantity of DNA was presumably due to the low cell density of *Sphingomonas* sp. ERG5 when grown in the relatively low nutrient medium R2A (compared to e.g. LB). A paired-end sequencing library for the Illumina was built with an average insert-size of 500 bp using a modified protocol for the NEBNext® Ultra™ DNA Library Prep Kit for Illumina® (New England BioLabs Inc). The library was loaded onto approximately 1/15 of an Illumina flow-cell and 2×100 base paired-end sequencing was performed on an Illumina® HiSeq 2000 machine.

### Genetic analysis

The raw reads were preprocessed using AdapterRemoval [15] and then assembled using the Velvet *de*-*novo* assembler (version 1.2.08) [16] with scaffolding switched off. An initial automated annotation was performed using the RAST service [17], while the sequences described in detail in this study were manually annotated using BLASTX with standard parameters [18] and Pfam domain search (version 27.0.) [19]. Open reading frames (ORFs) were predicted using the EasyGene server [20], [21]. A genetic map of plasmid pCADAB1 was generated using DNAplotter [22]. Copy numbers of genetic elements was estimated by comparing sequence coverages, as seen elsewhere [23].

The following programs and scripts were used for identification and analysis of IS-elements: ISfinder [24], einverted from the EMBOSS software package [25] and COILS [26]. A genetic map of transposon Tn6228 showing nucleotide similarities to gene clusters from *Sphingomonas* sp. 58-1 (accession no. AB353895.1), *Sphingomonas* sp. tfd44 (accession no. AY598949.1) and *Sphingobium herbicidovorans* MH (accession no. AJ628861.1) was constructed using Easyfig [27] with BLASTN implemented. The figure was edited for visual improvement in Inkscape [28].

### Phylogenetic analysis of CadA

The derived amino acid sequence of the *cadA* ORF from *Sphingomonas* sp. ERG5 was used as query for a BLASTX [18] search with standard parameters. Homologous sequences were chosen for analysis from the BLASTX hits with a cutoff value of 50% amino acid identity. The sequences were aligned using the Phylogeny.fr pipeline [29]: sequences were aligned with T-Coffee (v6.85) [30] using the pair-wise alignment methods Mlalign_id_pair and Mslow_pair. Following alignment, ambiguous regions were removed using Gblocks (v0.91b) [31] with the following parameters: minimum length of a block after gap cleaning: 10, no gap positions allowed in final alignment, all segments with contiguous nonconserved positions bigger than 8 were rejected, minimum number of sequences for a flank position: 85%. A phylogenetic tree was constructed using the Maximum Likelihood method and the Jones-Taylor-Thornton (JTT) amino acid substitution model [32], implemented in the PhyML program (v3.0 aLRT) [33], [34]. The reliability for internal branches was assessed using the bootstrapping method with 100 bootstrap replicates. The tree was visualized with MEGA4.0.2 [35]. Similar trees were constructed for CadBR and TfdBCDEFKR.

### PCR for identification of transposon position

**Figure 1:**
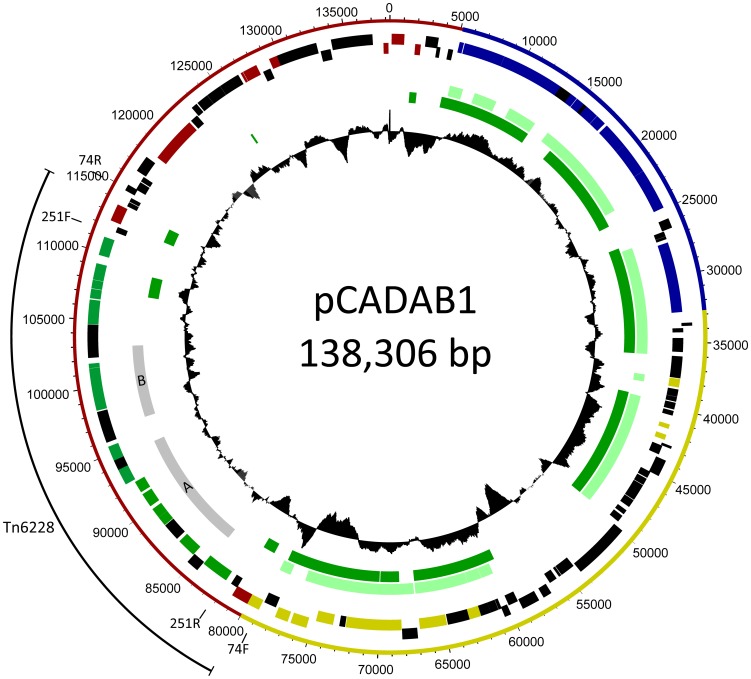
Genetic map of plasmid pCADAB1 and composite transposon Tn6228. The outer ring shows the functional regions of the plasmid: conjugative transfer (blue), plasmid stability and maintenance (yellow) and region containing multiple IS-elements (red). The composite transposon Tn6228 is displayed on the outside of the plasmid backbone (black line) Primers 74F, 74R, 251F and 251R for PCR have been marked on the outer ring. Inside of the backbone, predicted ORFs on either the positive strand (top blocks) or negative strand (bottom blocks) are displayed. Genes involved in degradation of MCPA are highlighted (green). Also highlighted are IS-elements (red), genes related to the type 4 secretion system (blue) and genes involved in plasmid maintenance and stability (yellow). The middle circle shows similarity to other sequences: Grey bars indicate collinear blocks of similarity to A) *Sphingomonas* sp. tfd44 (acc. no. AY598949.1) and B) *Sphingomonas* sp. 58-1 (acc. no. AB353895.1), while bright green bars indicate collinearity with plasmid pNL1 (acc. no. CP000676.1) and dark green with plasmid pCAR3 (acc. no. AB270530.1). The minimum nucleotide similarity in collinear blocks is 72%. The inner circle displays the G+C content (window size  = 1000, step size  = 10).

Primers for PCR amplification of DNA for capillary sequencing were designed using primer-BLAST [36] and synthesized by Eurofins MWG Operon (Ebersberg, Germany). All primers were designed to have a melting temperature (Tm) of approximately 63°C and are shown in Table 1. The primers were used in different combinations to test for 1) the presence/lack of transposon Tn6228 on plasmid pCADAB1, 2) circular intermediate state of Tn6228, 3) presence of Tn6228 in the pCADAB1 at 3′ end of transposon, 4) presence of Tn6228 in pCADAB1 at the 5′ end of transposon. The primed positions are shown in Figure 1 and the combinations of primers are shown in Table 2. PCRs were set up in 20 µl reactions containing 200 µM dNTPs, 0.5 µM of both forward and reverse primers, 0.2 µl of Phusion® High-Fidelity DNA Polymerase (New England Biolabs), 4 µl of 5× Phusion GC reaction buffer, 3% DMSO, 1 µl template DNA and up to 20 µl of Molecular Biology Grade Water (MOBIO). PCRs were performed on a S1000™ Thermal Cycler (BIO-RAD) using a Touch Down (TD) PCR program: an initial denaturation step at 98°C for 40 seconds followed by 15 cycles with denaturation at 98°C for 10 seconds, annealing starting at 72°C for 15 seconds and lowered by 1°C per cycle, elongation at 72°C for 80 seconds. After TD cycling, the protocol continued with 20 cycles of 98°C for 10 seconds, 57°C for 15 seconds, 72°C for 80 seconds and a final elongation step of 72°C for 5 minutes. TD-PCR products were run on a 1% agarose gel, which was cast using modified TAE-buffer from the Millipore DNA Gel Extraction Kit and containing 0.5 µg/ml ethidium bromide. The amplification products were excised from the gel using Millipore DNA Gel Extraction Kit and DNA concentrations were measured using the Qubit® 2.0 Fluorometer (Invitrogen). The extracted PCR products were shipped to Macrogen for capillary sequencing.

**Table 1:** Primers used for TD-PCR.

Primer	Type	Target region	Sequence (5′-3′)	Anneal temp (°C)
**251F**	Forward primer	5′ end of transposon	AACCGGAACCCATCGGCATC	62.89
**251R**	Reverse primer	3′ end of transposon	CGGAACCGCTGCTCCATACC	63.25
**74F**	Forward primer	Upstream of transposon	CACCTTGACCACGGGTTCGG	63.33
**74R**	Reverse primer	Downstream of transposon	CCAAAGCGGTATCTGCCGTGA	63.02

**Table 2:** Combination of primers for TD-PCR and tested hypotheses.

Primerset (primers used)	Tested hypothesis	Predicted fragment size (bp)
**P1 (74F+74R)**	The plasmid only contains the transposase, not the transposon	3373
**P2 (251F+251R)**	The transposon has a circular state	3174
**P3 (251F+74R)**	The transposon is located on the plasmid in the predicted position. This primerset primes in the 3′-end of transposon	3333
**P4 (74F+251R)**	The transposon is located on the plasmid in the predicted position. This primerset primes in the 5′-end of transposon	3234

### Nucleotide sequence accession numbers

The nucleotide sequence of plasmid pCADAB1 has been submitted to the GenBank database under accession number KF494257.

## Results

### Shotgun sequencing of *Sphingomonas* sp. ERG5

The MCPA-degrading *Sphingomonas* sp. ERG5 was sequenced using the Illumina HiSeq2000 platform. Preprocessing and assembly of 12,540,301 paired reads yielded 69 contigs larger than 500 bp. These contigs have a combined size of 5.7 Mb, a G+C content of 63.7% and an average coverage of approximately 91×. The 16S gene sequence of *Sphingomonas* sp. ERG5 showed 99% similarity to *Sphingomonas alpina* strain S8-3 (accession no. GQ161989.1).

A single homolog of the TfdA-related taurine catabolism dioxygenase (TauD) was found in the chromosomal contigs; however, the deduced amino acid sequence of this gene showed little similarity to any verified TfdA proteins. The closest TfdA 2,4-D dioxygenase homolog to this gene is that of *Ralstonia solanacearum* CMR15 (accession no. YP_008997670.1), which is only 31% identical to the taurine dioxygenase of *Sphingomonas* sp. ERG5.

However, genes homologous to *cadAB* were located on a 32.5 kbp contig containing a 1266 bp IS3 element. This contig had a G+C content of 62.4% and a coverage of 106.5×. An identical copy of the IS3 element was present in an approximately 106 kbp circular contig (circularity and size confirmed by PCR - data not shown) with a coverage of 97.5× and a G+C content of 63.4%, which contained genes involved in conjugative plasmid transfer, indicating that this contig represents a conjugative plasmid (pCADAB1). The total coverage of the IS3 elements was 199.8×, indicating that this insertion sequence has a copy number of approximately 2. It was hypothesized that the 32.5 kbp contig actually represents a composite transposon (Tn6228), flanked by identical, directly oriented copies of the IS3 elements, which is situated on the conjugative plasmid contig (review on composite transposons [37]). The length of the IS3 elements is longer than the insert-size of the paired-end Illumina sequencing, which prevents the Velvet program from correctly assembling this repeated structure as summarized by [38].

### Genetic organization of transposon Tn6228 and plasmid pCADAB1

To investigate the possible position of Tn6228 in plasmid pCADAB1, PCRs were performed (Table 1+2 and Figure 1), followed by sequencing of the PCR products (results not shown). Primer set 1 was designed so that a 3373 bp product would result from the PCR if only the IS3 element is present and not the entire Tn6228. PCR with primer set 2 will yield a 3174 bp product if Tn6228 has a circular state outside pCADAB1, since primers 251F and 251R target the sequence at either end of Tn6228 and prime outwards. Primer sets 3 and 4 use combinations of primers targeting either Tn6228 or pCADAB1 sequence near the IS3 elements. PCR products from these primer sets with the predicted sizes (Table 2) will indicate that Tn6228 is situated on pCADAB1 in the predicted location. The PCR products had the expected sizes, except from that of primer set 1 which was larger than 3373 bp. The bands were excised from the gel and the PCR products were sequenced. The sequencing revealed that PCR using primer sets 3 and 4 had amplified DNA from the predicted location of Tn6228, confirming the predicted position on pCADAB1. The PCR using primer set 2 yielded a product with the predicted size, and sequencing of the PCR product confirmed that Tn6228 indeed has a circular state, as well as linearly inserted into pCADAB1. The product from the PCR with primer set 1 could not be sequenced properly, yielding only base calls of poor quality.

The organization of pCADAB1 with Tn6228 in the determined position is shown in Figure 1. Here, predicted genes, G+C content and regions of homology are shown. With PCR and PCR product sequencing it was confirmed that the transposon exists in a circular state, as well as in a linear state in the plasmid which is typical for IS3 elements [39] (reviewed by [40]). Identical copies of the IS3 element were determined to be flanking the composite transposon, when inserted into the plasmid. The IS3-like transposase found here contained a leucine-zipper motif, a DDE-motif and imperfect inverted repeats - all of which are expected to be present in a functional IS3-element [41].

### Annotation of genes in transposon Tn6228

**Figure 2:**
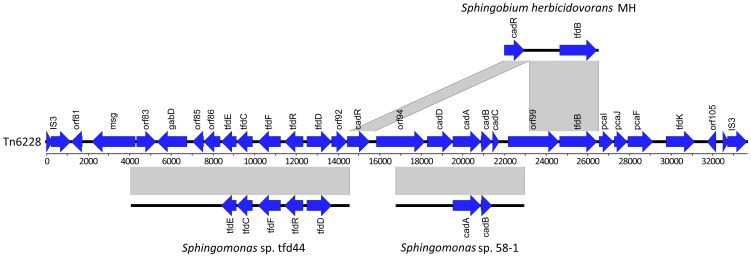
Genetic organization of the composite transposon Tn6228 and comparison to other gene clusters. Genes are marked with blue arrows. Gene abbreviations are *msg*: malate synthase G, *gabD*: putative succinate-semialdehyde dehydrogenase, *tfdE*: dienelacatone hydrolase, *tfdC*: chlorocatechol 1,2-dioxygenase, *tfdF*: maleylacetate reductase, *tfdR*: LysR family transcriptional regulator, *tfdD*: chloromuconate cycloisomerase, *tauE/safE*: sulfite exporter, *cadR*: AraC family transcriptional regulator, *cadD*: oxidoreductase component of Rieske non-heme iron oxygenase (RO), *cadA*: large subunit of 2,4-D oxygenase, *cadB*: small subunit of 2,4-D oxygenase, *cadC*: ferredoxin component of RO, *tfdB*: dichlorophenol hydroxylase, *pcaI*: 3-oxoacid CoA-transferase A subunit, *pcaJ*: 3-oxoacid CoA-transferase B subunit, *pcaF*: beta-ketoadipyl CoA thiolase, *tfdK*: transport protein. Nucleotide similarity is shown as gray bars linking to the corresponding gene clusters in *Sphingomonas* sp. tfd44 (acc. no. AY598949.1), *Sphingomonas* sp. 58-1 (acc. no. AB353895.1) and *Sphingobium herbicidovorans* MH (acc. no. AJ628861.1). Nucleotide similarities are shown with a cutoff value of 98% and were identified with BLASTN as implemented in Easyfig [27]. The composite figure shown here was compiled in Inkscape [28].

EasyGene ORF prediction of the transposon contig yielded 27 ORFs, which are numbered relative to plasmid start in the following sections. Manual annotation using BLASTX and Pfam domain search resulted in the predicted genes shown in Table S1 and Figure 2.

Within the Tn6228 transposon, three different gene clusters showed high similarity to previously identified clusters of 2,4-D catabolic genes (Figure 1 and 2). The first, 10493 bp in length, spans transposon-relative positions 2894–13386, contains 10 predicted ORFs and shares high nucleotide similarity (99% identity) to a chlorocatechol catabolic gene cluster from *Sphingomonas* sp. tfd44 [42] (accession no. AY598949.1). Due to sequence similarity, as well as BLASTX and Pfam results, ORFs 83–92 in this gene cluster were designated to encode putative LysR-type regulator, putative succinate-semialdehyde dehydrogenase GabD, hypothetical protein, hypothetical protein with Rieske domain, dienelactone hydrolase TfdE, chlorocatechol 1,2-dioxygenase TfdC, maleylacetate reductase TfdF, LysR-type regulator TfdR, muconate lactonizing enzyme TfdD and putative Tau/SafE sulfite exporter, respectively. The second gene cluster is 6182 bp in length and is located 2212 bp downstream of the first cluster in transposon-relative positions 15599–21780. It shows high nucleotide similarity (98% identity) to a clone fragment from *Sphingomonas* sp. 58-1 [8] (accession no. AB353895.1) and contains ORFs 95–98, which are annotated to encode pyridine nucleotide-disulphide oxidoreductase, large subunit of 2,4-D oxygenase CadA, small subunit of 2,4-D oxygenase CadB, and putative 2-hydroxybenzoate 5-hydroxylase ferredoxin CadC, respectively. Between the gene clusters, ORFs 93 and 94 encode transcriptional regulator CadR and a putative TonB-dependent receptor, respectively. Upstream of the first gene cluster, ORFs 81 and 82 encode an unknown hypothetical protein and a malate synthase G, respectively. Downstream of the second gene cluster, ORFs 99–107 were predicted to encode a putative TonB-dependent receptor, dichlorophenol hydroxylase TfdB, A and B subunits of a 3-oxoacid CoA-transferase PcaIJ, a beta-ketoadipyl CoA thiolase PcaF, transport protein TfdK, a putative diguanylate cyclase and two ORFs constituting a transposase of the IS3 family. The genes *cadR*, *orf99* and *tfdB* showed high nucleotide similarity with a gene cluster from *Sphingobium herbicidovorans* MH (accession no. AJ628861.1), although *orf99* might be truncated in this bacterium (Figure 2).

**Figure 3:**
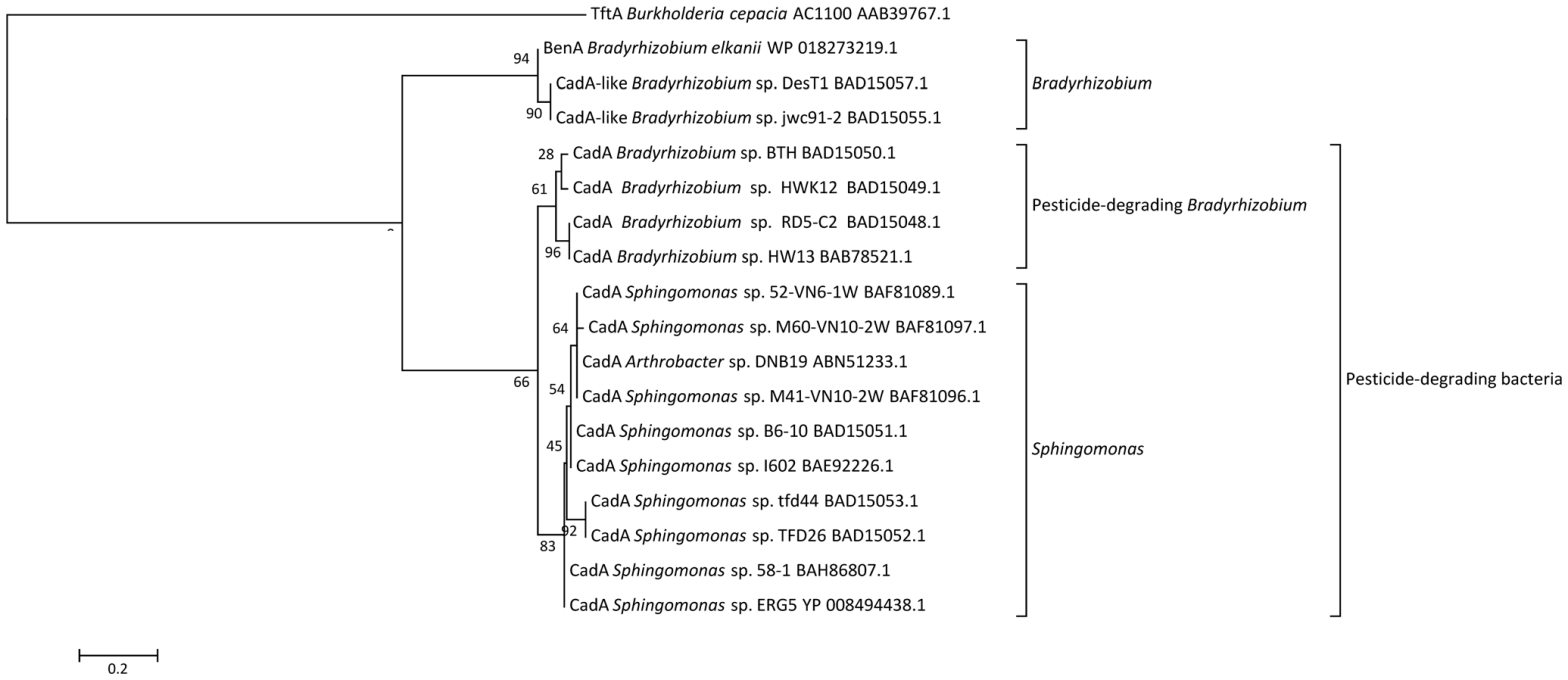
Dendrogram representing the phylogenetic relationship of representative CadA sequences. Representative sequences for alignment were identified from a BLASTX search using the derived amino acid sequence of CadA from *Sphingomonas* sp. ERG5 as query. BLASTX hits were picked for comparison with a cutoff value of 50% for amino acid identities. The support of each branch, as determined from 100 bootstrap samples, is indicated as a percentage at each node. Sequences were aligned with T-Coffee (v6.85) [30] and the alignment was curated with Gblocks (v0.91b) [31]. A phylogenetic tree was constructed using a maximum likelihood approach and the JTT substitution model [32] in the PhyML program (v3.0 aLRT) [33], [34]. The resulting unrooted phylogenetic tree was visualized in MEGA4.0.2[35].

A phylogenetic tree of 18 selected CadA amino acid sequences from the genera *Sphingomonas*, *Bradyrhizobium, Burkholderia* and *Arthrobacter* was constructed (Figure 3). This tree shows a clustering of CadA, similar to a clustering previously described [5], where CadA can be separated into 3 different clades: a *Sphingomonas* clade, a 2,4-D degrading *Bradyrhizobium* clade and a clade containing *Bradyrhizobium* not degrading 2,4-D. The *Sphingomonas* clade also contains a single member of the *Arthrobacter* genus, which has possibly acquired the *cadA* gene through horizontal gene transfer (HGT).

### Properties of plasmid and transposon

EasyGene predicted 110 ORFs in the plasmid pCADAB1, and an automated annotation by RAST revealed that the predicted genes could be divided into cohesive regions of function: conjugal transfer, plasmid maintenance/stability and a region containing various IS-elements (Figure 1). The regions for conjugal transfer and maintenance/stability showed similarity in their nucleotide sequences to the sphingomonad plasmids pCAR3 (accession no. AB270530.1) and pNL1 (accession no. CP000676.1), which have been shown to harbor the genes involved in degradation of xenobiotics [43], [44]. Within the conjugal transfer region, the genes *traALEKBVCFWUNFHGID*, *trbC* and *dsbC* encode a type 4 secretion system (T4SS) for conjugative transfer. These genes are very similar to those of pNL1 (Figure 1), which has been shown to be capable of conjugative transfer [44], [45]. The maintenance/stability region of pERG5 contains genes encoding plasmid replication and partitioning systems, a toxin-antitoxin system, a multimer resolution system and restriction-modification systems.

The transposon was assembled by Velvet as one contig at a size of approximately 32.5 kbp, a G+C content of 62.4% and a coverage of 106.5×. Based on the average sequence coverages of the transposon, plasmid and chromosome, it was estimated that the transposon copy number is 1.17 relative to the chromosome and 1.09 relative to the plasmid. This approach for estimating copy numbers has been utilized previously [23]. Two ORFs (ORF 106–107) were predicted to constitute an IS-element gene, which was confirmed by ISfinder [24] and showed 84% identity with the IS3 family (group IS407) transposase ISSpma1 from *Sphingopyxis macrogoltabida* (accession no. AB196775). The IS-element has imperfect inverted repeats of 49 bp (32/49) (IRL: TGATCTGCCCCCTTCTGAGTGGTCCAAAATCTCTGTTATTTTGGATCAT and IRR: ACAATGCACTAACAATCAACACGGACCACTCAGTGGGGGCCGGTCA). Furthermore, a leucine-zipper motif, involved in dimer formation of the transposase gene [41], was identified (data not shown) using the COILS program [26]. The conserved transposase-related DDE motif [41] was manually identified in the derived amino acid sequence as Dfv-55-sDngse-31-Esfngslr.

## Discussion

In this study, we have identified a composite transposon (Tn6228) containing the complete genetic blueprint for degradation of 2,4-D and MCPA in a *Sphingomonas* sp. recently isolated from MCPA-degrading bacterial communities that originated from a herbicide-impacted groundwater aquifer [14]. This transposable element, which contains the catabolic genes cadAB in a novel genetic context, was situated on a conjugative plasmid. The plasmid contig, approximately 106 kbp in size, contains a single, perfectly identical copy of the IS3-element also present in the transposon contig. It was hypothesized that transposon Tn6228 would exist on the plasmid pCADAB1 flanked by identical IS3-elements, as well as in a circular intermediate state, typical of IS3-elements [39]. Due to the length of the transposase (1266 bp) being longer than the insert length in the Illumina library (500 bp), the assembler will not be able to solve the structure shown in Figure 1, where the transposon is inserted into the plasmid with IS3 elements flanking it. The structure displayed in Figure 1 was confirmed with PCR and product sequencing, using combinations (Table 2) of the primers shown in Figure 1 and Table 1. The PCR product sequencing from PCR primer set 1 did not provide any eligible sequence, while sequencing of the products from PCRs 3 and 4 (Table 2) confirmed that the transposon is situated on pCADAB1 (data not shown). The failure of PCR and product sequencing with primer set 1 indicates that a scenario with only an IS3 element and not Tn6228 in the predicted position is not plausible. The fact that the catabolic genes are located on a conjugative plasmid is in good agreement with previous observations in xenobiotic-degrading sphingomonads [45].

### Catabolic genes involved in degradation of MCPA

The well-studied t*fdAα* gene could not be found in the assembled contigs of *Sphingomonas* sp. ERG5. However, the alternative Rieske non-heme iron oxygenase (RO) *cadAB* genes were found within the composite transposon Tn6228 also harboring the remaining genes in the degradation pathway of 2,4-D and related compounds [42]. The 1347 bp *cadA* ORF shows very high amino acid similarity (99% identity) to the α-subunit of 2,4-D oxygenase from *Sphingomonas* sp. 58-1 (accession no. BAH86807.1) and contains a Rieske motif and a catalytic domain, typical of RO enzymes. The 567 bp *cadB* ORF also shows high amino acid similarity (98% identity) to CadB from *Sphingomonas* sp. 58-1. β-subunits of RO oxygenases have been reported to be involved in substrate specificity [46].

In three-component RO-systems, a reductase receives electrons from NAD(P)H which is carried by a ferredoxin to the catabolic oxygenase, as reviewed in [47]. *orf95* is predicted to encode a putative oxidoreductase protein. This ORF shows high similarity to *orf*1 from *Sphingomonas* sp. 58-1 (98% identity). *orf98* is predicted to encode a Rieske ferredoxin component in the Rieske non-heme iron oxygenase (RO) family [48] and shows highest similarity (54% identity) to a 2-hydroxybenzoate 5-hydroxylase ferredoxin from *Achromobacter piechaudii* HLE (accession no. ZP_15933011.1). Even though all the parts of a three-component RO-system seem to be present in *Sphingomonas* sp. ERG5, the roles of the ferredoxin and oxidoreductase components in this RO system remain unknown, since it has previously been shown that the *cadAB* genes were necessary for conversion of 2,4-D to 2,4-DCP, but the oxidoreductase, putatively encoded by *orf95*, was not [8]. A ferredoxin component was not identified in the paper describing *Sphingomonas* sp. 58-1 [8], even though the sequence for a ferredoxin is present in the clone fragment in its GenBank entry (accession no. AB353895.1). The *cadABC* genes were first discovered in the strain *Bradyrhizobium* sp. HW3. Here it was shown that the ferredoxin component encoded by *cadC* indeed was essential for 2,4-D transformation, but no reductase was identified [2]. The finding of possible ferredoxin and reductase components of a three-component RO system in *Sphingomonas* sp. ERG5 suggests that these might be involved in the first catabolic step in 2,4-D and MCPA degradation, leading to the designation of putative *cadC* and putative novel gene *cadD* of the ferredoxin and reductase *orf*s, respectively.

Following the initial aryl ether linkage cleavage of MCPA and 2,4-D catalyzed by CadAB, the subsequent catabolic and regulatory pathway are represented by 8 ORFs in Tn6228: *tfdB, tfdC, tfdD, tfdE, tfdF, tfdK, tfdR* and *cadR*. These ORFs showed high amino acid similarity to previously described genes in the chlorophenoxyacetic acid metabolic pathway of strains *Sphingobium herbicidovorans* and *Sphingomonas* sp. TFD44 [42], [49] (Table S1). The *tfd*-genes are involved in the *ortho* cleavage pathway of 2,4-D and related compounds and were first studied in *Cupriavidus pinatubonensis* JMP134 [50]. The *tfdB* gene encodes a dichlorophenol hydroxylase, which converts 4-chloro-2-methylphenol (MCP) to 5-chloro-3-methyl-catechol (MCC), which is then converted to chloromuconate by a chlorocatechol 1,2-dioxygenase, encoded by *tfdC*. The product of *tfdD* then transforms chloromuconate to dienelactone. Finally, dienelactone is converted first to maleylacetate then to 3-oxoadipate by the gene products of *tfdE* and *tfdF*, respectively.

Additionally, *orf101* and *orf102* encode subunits A and B of a 3-oxoadipate CoA-transferase, enabling the reaction yielding 3-oxoadipyl-CoA and succinate from 3-oxoadipate and succinyl-CoA. The 3-oxoadipate for this reaction could be provided by the reaction catalyzed by TfdF. *orf103* is predicted to encode a 3-oxoadipyl CoA thiolase that can convert 3-oxoadipyl-CoA to succinyl-CoA and acetyl-CoA, both of which are further degraded the tricarboxylic acid cycle (TCA) [51]. *orf82* and *orf84* are designated to encode a malate synthase G and a putative succinate-semialdehyde dehydrogenase, respectively. Both of these enzymes are involved in the TCA and glyoxylate cycle; however homologs of these proteins were identified on chromosomal contigs (data not shown), suggesting that these plasmid-encoded enzymes are not essential for the host cell. Malate synthase G can convert acetyl-CoA into malate and succinate-semialdehyde dehydrogenase is possibly involved in the further degradation of succinyl-CoA.

In addition to the catabolic genes, regulatory genes *cadR* and *tfdR* as well as a gene encoding the transport protein TfdK are also present within the transposon. The deduced amino acid sequence of the *cadR* gene found here is 100% identical to that of *Sphingobium herbicidovorans* MH (accession no. CAF32814.1) and has an identity of 62% to the *cad* gene transcriptional regulator from *Bradyrhizobium* sp. HW13 (accession no. BAB78520.1). The *cadR* gene was found to be essential for 2,4-D degradation in *Bradyrhizobium* sp. HW13 and it was hypothesized, due to sequence similarity, that *cadR* encodes a positive transcriptional regulator [2].

*orf90* was annotated as a LysR-type transcriptional regulator TfdR with high similarity (99% identity) to TfdR of *Sphingomonas* sp. TFD44 (accession no. AAT99366.1). It has been suggested that the transcriptional inducer of the 2,4-D pathway is the product of the TfdC protein, 2,4-dichloromuconate [52].

A homologue of the *tfdK* gene in *Sphingobium herbicidovorans* MH (accession no. CAF32820.1) encoding a protein of the major facilitator superfamily (MFS) was found to be encoded by *orf104*. In *Cupriavidus pinatubonensis* JMP134 it was shown that TfdK is an active transporter of 2,4-D and increases its uptake rate of 2,4-D up to 10 times compared to a tfdK mutant [53].

### Evolution of genetic elements

Two of the three gene clusters in Tn6228 previously described, showed high degrees of collinearity and sequence similarity to previous isolates [8], [42]. This could indicate that this specific genetic organization of the *cad-* and *tfd-*genes is highly conserved within sphingomonads capable of PAA-herbicide degradation or that these genes are an integral part of the genetic repertoire of sphingomonads. It has previously been discussed that sphingomonads harbor large numbers of mobile genetic elements [54] and that these are likely responsible for regular genetic exchange of biodegradative potential between sphingomonads [55]. The sequence conservation displayed in Figure 2 strongly supports that these catabolic enzymes are subject to HGT between sphingomonads. The non-collinearity between *Sphingomonas* sp. ERG5 and the gene cluster from *Sphingobium herbicidorans* MH could indicate that genetic rearrangements have occurred in one of the strains (Figure 2). The G+C values of Tn6228 shows that no internal regions with a markedly different G+C content are present within the transposon (Figure 1). However, when comparing with the plasmid backbone of pCADAB1, the region containing mobile genetic elements (including Tn6228) has a tendency towards slightly lower G+C contents. This suggests that the region with many mobile genetic elements might contain genes with an origin different from that of the plasmid backbone. Furthermore, the average G+C contents of pCADAB1 and Tn6228 are not different from that of the chromosomal contigs, suggesting that either these genetic entities were not recently acquired via horizontal gene transfer or that they originate from a bacteria with similar G+C content. Despite *Sphingomonas* sp. 58-1 being isolated in Japan, *Sphingomonas* sp. TFD44 being isolated in North America and *Sphingomonas* sp. ERG5 being isolated in Denmark, these strains show remarkable homology at the DNA level in the catabolic genes. All these were isolated from pesticide-polluted soils, and it would be interesting to attempt the isolation of *cadAB*-harboring sphingomonads from pristine environments for molecular comparisons in order to study the origin of this PAA degradation pathway initiated by the *cad*-genes.

Considering the phylogenetic separation of the CadA amino acid sequences shown in Figure 3 into a *Sphingomonas* and two *Bradyrhizobium* clades, it seems plausible that there exist multiple (at least three) classes of CadA that are each inherent to only the *Sphingomonas* or *Bradyrhizobium* genus. It was suggested that the *Sphingomonas* cluster and 2,4-D degrading *Bradyrhizobium* cluster had evolved from a shared ancestral gene, indicating different evolution of the *cadA* gene in 2,4-D degraders from that of non-2,4-D degraders [5]. Similar phylogenetic trees were constructed for the genes *tfdBCDEFRK* and *cadR*. The trees for *tfdBD* (Figures S1 and S2) contain monophyletic clades of the *Sphingomonadaceae* family, indicating that these genes are more similar within this family than with other phylogenetic groups (e.g. the *Bradyrhizobiaceae* family or *Burkholderiales* order). This suggests that parts of the 2,4-D-degradation pathway has evolved separately in the *Sphingomonadaceae* family, which is supported by others [9] who hypothesized that the *tfdA*α gene has evolved distinctly in sphingomonads and *Bradyrhizobium* without horizontal gene transfer. However, representatives of the downstream *tfd*-genes from *Sphingomonadaceae* are not yet abundant enough in the databases for proper evolutionary studies. The remaining phylogenetic trees suggest that horizontal gene transfer has had an effect on the evolution of the *tfdCEFRK* genes since no clear monophyletic groups are defined for any particular genera (data not shown).

The plasmid backbone of pCADAB1, encoding genes related to conjugative transfer and plasmid stability, showed high similarity and collinearity to plasmids pCAR3 (accession. no. AB270530.1) and pNL1 (accession. no. CP000676.1) from *Sphingomonas* sp. KA1 and *Novosphingobium aromaticivorans* DSM12444, respectively (Figure 1). Plasmid pNL1 has been shown to be able to transfer to other sphingomonads [45], and both pNL1 and pCAR3 have been suggested to be members of a novel incompatibility group of plasmids [43]. Though additional mating experiments should be carried out on this putatively novel incompatibility group, preliminary investigations on the plasmid replication and partitioning genes *repA* and *parA* of pCADAB1 indicates that this plasmid belongs to this group (data not shown). In fact, the backbones of the described plasmids are similar and collinear, while the region containing IS-elements and transposons is completely unrelated, suggesting that these mobile genetic elements might serve as the tools for acquiring new catabolic genes while the backbone is the unchangeable toolbox of these sphingomonad strains. It has been shown that many sphingomonad strains contain degradative plasmids [45]. If these plasmids are restricted to reside in members of the sphingomonad genera, it could explain why genes such as *cadA* evolve separately from other *cadA*-harboring genera. Further studies on the plasmids of sphingomonads will possibly provide insight to the degradative capabilities of these bacteria and why they apparently are successfully thriving in polluted environments.

## Conclusions

In this study, a composite transposon was identified which harbors the complete set of genes for MCPA and 2,4-D degradation, including initial steps of the TCA cycle and regulatory genes. The transposon was furthermore found to be situated on a putatively conjugative plasmid. The pathway initiates with the CadAB Rieske non-heme iron monooxygenase, rather than the α-ketoglutarate-dependent dioxygenase TfdA enzyme, that is most often associated with this pathway.

## Supporting Information

Table S1 **Predicted ORFs with annotations and putative protein properties.**
(DOCX)

Figure S1 **Dendrogram representing the phylogenetic relationship of representative TfdB sequences.**
(TIF)

Figure S2 **Dendrogram representing the phylogenetic relationship of representative TfdD sequences.**
(TIF)
