# Supplementary material for: Novel Insight into the Genetic Context of the cadAB Genes from a 4-chloro-2-methylphenoxyacetic Acid-Degrading Sphingomonas
Source: PLoS One. 2013 Dec 31;8(12):e83346. doi: 10.1371/journal.pone.0083346 (PMC3877037; doi:10.1371/journal.pone.0083346)
Supplement: Table S1 — Predicted ORFs with annotations and putative protein properties. (DOCX) [file pone.0083346.s001.docx]

Table S1. Predicted ORFs with annotations and putative protein properties. The ORF numbering is according to plasmid pCADAB1 start, while the position coordinates are relative to transposon start.

| ORF no. | Coding sequence | Position (nt) | Strand | Protein length (amino acids) | Pfam motifs/Function | Amino acid identity/similarity (%) to closest homolog | | Accession no. of homolog |
| --- | --- | --- | --- | --- | --- | --- | --- | --- |
| *orf79;* Manually inserted due to experimental evidence | Transposase *orfA* | 64-339 | + | 92 | HTH Tnp 1; transposase | 96/97; transposase from *Sphingomonas* sp. PAMC 26605 | WP_010184853.1 | |
| *orf80*; Manually inserted due to experimental evidence | Transposase *orfB* | 288-1232 | + | 315 | HTH 21; integrase core domain | 92/95; transposase from *Granulibacter* *bethesdensis* CGDNIH1 | YP_745620.1 | |
| *orf81* | Hypothetical protein | 1250-1786 | - | 179 | No Pfam domain/conserved hypothetical protein | 46/65; unknown from *Acinetobacter* *haemolyticus* ATCC 19194 | EFF81335.1 | |
| *orf82* | Malate synthase G | 2240-4327 | - | 696 | Malate synthase | 80/88; Malate synthase G from *Sphingomonas* *japonicum* | WP_006959691.1 | |
| *orf83* | Putative LysR-type regulator | 4393-5322 | + | 310 | HTH, LysR substrate binding domain; uncharacterized LysR transcriptional regulator | 100/100; putative LysR-type regulator from *Sphingomonas* sp. tfd44 | AAT99360.1 | |
| *orf84* | *gabD* | 5368-6807 | - | 480 | TIGR01780: SSADH succinate-semialdehyde dehydrogenase | 100/100; putative aldehyde dehydrogenase from *Sphingomonas* sp. tfd44 | AAT99361.1 | |
| *orf85* | Hypothetical protein | 7067-7603 | - | 179 | No Pfam domain | 98/98; unknown from *Sphingomonas* sp. tfd44 | AAT99362.1 | |
| *orf86* | Hypothetical protein with Rieske domain | 7600-8397 | - | 266 | Rieske; hypothetical protein | 99/98; unknown from *Sphingomonas* sp. tfd44 | AAT99362.1 | |
| *orf87* | *tfdE* | 8449-9171 | - | 241 | DLH; Dienelactone hydrolase | 100/100; dienelactone hydrolase from *Sphingobium* *herbicidovorans* | CAF32819.1 | |
| *orf88* | *tfdC* | 9184-9951 | - | 256 | Dioxygenase C; chlorocatechol 1,2-dioxygenase | 99/100; *tfdC* from *Sphingomonas* sp. tfd44 | AAT99364.1 | |
| *orf89* | *tfdF* | 10218-11297 | - | 360 | Fe-ADH; Maleylacetate reductase | 100/100; *tfdF* from *Sphingomonas* sp. tfd44 | AAT99365.1 | |
| *orf90* | *tfdR* | 11484-12374 | - | 297 | HTH, LysR substrate binding domain; LysR-type regulator involved in catechol metabolism and activation of tfdA and tfdB | 99/100; *tfdR* from *Sphingomonas* sp. tfd44 | AAT99366.1 | |
| *or91* | *tfdD* | 12554-13750 | + | 399 | MR MLE N, MR MLE C; muconate lactonizing enzyme(MLE) | 96/98; tfdD from *Sphingomonas* sp. tfd44 | AAT99367.1 | |
| *orf92* | Putative *tauE*/*safE* sultite exporter | 13747-14493 | + | 249 | TauE; Sulfite exporter TauE/SafE | 37/56; hypothetical protein RLO149_c007770 from *Roseobacter* *litoralis* Och 149 | YP_004689767.1 | |
| *orf93* | *cadR* | 14490-15572 | + | 361 | HTH18; transcriptional regulator | 100/100; transcriptional regulator from *Sphingobium* *herbicidovorans* | CAF32814.1 | |
| *orf94* | Putative TonB-dependent receptor | 15890-18226 | + | 779 | TonB dep Rec; TonB-dependent receptor | 48/61; TonB-dependent receptor from *Sphingomonas* sp. SKA58 | CAF32814.1 | |
| *orf95* | *cadD* | 18328-19566 | + | 413 | Pyr redox 2, Pyr redox, Reductase C-terminal; phenylpropionate dioxygenase ferredoxin reductase subunit | 98/98; hypothetical protein from *Sphingomonas* sp. 58-1 | ZP_01303298.1 | |
| *orf96* | *cadA* | 19563-20909 | + | 449 | Ring hydroxyl A; benzoate 1,2-dioxygenase, large subunit | 99/99; large subunit of 2,4-D oxygenase from *Sphingomonas* sp. 58-1 | BAH86807.1 | |
| *orf97* | *cadB* | 20909-21442 | + | 178 | Ring hydroxyl B; small subunit of phenylpropionate dioxygenase (or benzoate 1,2-dioxygenase, small subunit) | 98/98; small subunit of 2,4-D oxygenase from *Sphingomonas* sp. 58-1 | BAH86808.1 | |
| *orf98* | *cadC* | 21461-21790 | + | 110 | Rieske; Rieske non-heme iron oxygenase RO | 54/68; 2-hydroxybenzoate 5-hydroxylase ferredoxin from *Achromobacter* *piechaudii* HLE | ZP_15933011.1 | |
| *orf99* | Putative TonB-dependent receptor | 22215-24650 | + | 812 | TonB dep Rec; CirA Outer membrane receptor protein, mostly Fe transport | 97/98; outer membrane receptor protein from *Sphingobium* *herbicidovorans* | CAF32815.1 | |
| *orf100* | *tfdB* | 24677-26476 | + | 600 | FAD binding 3; hypothetical protein | 99/99; dichlorophenol hydroxylase from *Sphingobium herbicidovorans* | CAF32816.1 | |
| *orf101* | *pcaI* | 26576-27262 | + | 229 | CoA trans, 3-oxoacid CoA-transferase A | 86/95; 3-oxoacid CoA-transferase, A subunit from *Novosphingobium* sp. AP12 | ZP_10745725.1 | |
| *orf102* | *pcaJ* | 27301-27930 | + | 210 | CoA trans, 3-oxoacid CoA-transferase B | 89/94; 3-oxoacid CoA-transferase, B subunit from *Sphingomonas* sp. LH128 | ZP_10875128.1 | |
| *orf103* | *pcaF* | 27955-29160 | + | 402 | Thiolase N/C; | 86/93; beta-ketoadipyl CoA thiolase from *Sphingobium* *chlorophenolicum* L-1 | YP_004555586.1 | |
| *orf104* | *tfdK* | 29791-31164 | + | 458 | MFS 1; benzoate transport | 90/94; transport protein (*tfdK*) from *Sphingobium* *herbicidovorans* | CAF32820.1 | |
| *orf105* | Putative diguanylate cyclase | 31755-32180 | - | 142 | EAL; signal protein | 76/83; diguanylate cyclase domain protein from *Sphinogmonas* sp. S17 | ZP_08386810.1 | |
| Not predicted by EasyGene. Possibly a truncated gene. | Putative resolvase | 30973-31172 | + | 67 | No Pfam domain; resolvase | 89/93; resolvase from *Hyphomicrobium* sp. MC1 | YP_004677122.1 | |
| *orf106* | Transposase *orfA* | 32509-32784 | + | 92 | HTH Tnp 1; transposase | 93/96; transposase from *Sphingomonas* sp. PAMC 26605 | WP_010184853.1 | |
| *orf107* | Transposase *orfB* | 32733-33677 | + | 273 | HTH 21; integrase core domain | 92/95; transposase from *Granulibacter* *bethesdensis* CGDNIH1 | YP_745620.1 | |
